# Management of anomalous origin of right coronary artery from left coronary sinus

**DOI:** 10.1186/s13019-023-02354-7

**Published:** 2023-09-26

**Authors:** Tobin Mangel, Aabha Divya, Ravi De Silva

**Affiliations:** Cardiothoracic Surgery, Royal Papworth Hospital NHS Foundation Trust, Cambridge, UK

## Abstract

Aberrant origin of coronary artery is a rare congenital anomaly associated with increased risk of myocardial ischemia and sudden death, with the highest risk lesions being those arising from the opposite sinus of Valsalva. We report a case with an aberrant right coronary artery arising superior to the left coronary cusp, with a slit-like ostium, having an inter-arterial and intramural course through the aortic root, that underwent repositioning of the right coronary artery. We believe such cases warrant surgical correction and reimplantation is a safe, effective and reproducible technique.

## Introduction

Aberrant origin of coronary artery is a rare congenital anomaly associated with increased risk of myocardial ischemia and sudden death, with the highest risk lesions being those arising from the opposite sinus of Valsalva.

## Case report

A 68-year-old woman, had been troubled by intermittent chest pain for the last ten years, progressively worsening over the last few months with occasional palpitations. Further investigations revealed reversible ischaemia in the territory of the right coronary artery. CT scanning confirmed the diagnosis and demonstrated an acute-angled right coronary artery with a narrow orifice, originating superior to the left coronary sinus with an intramural, inter-arterial course between the aorta and pulmonary trunk, at the level of the pulmonary valve, resulting in moderate narrowing.

**Fig. 1:**
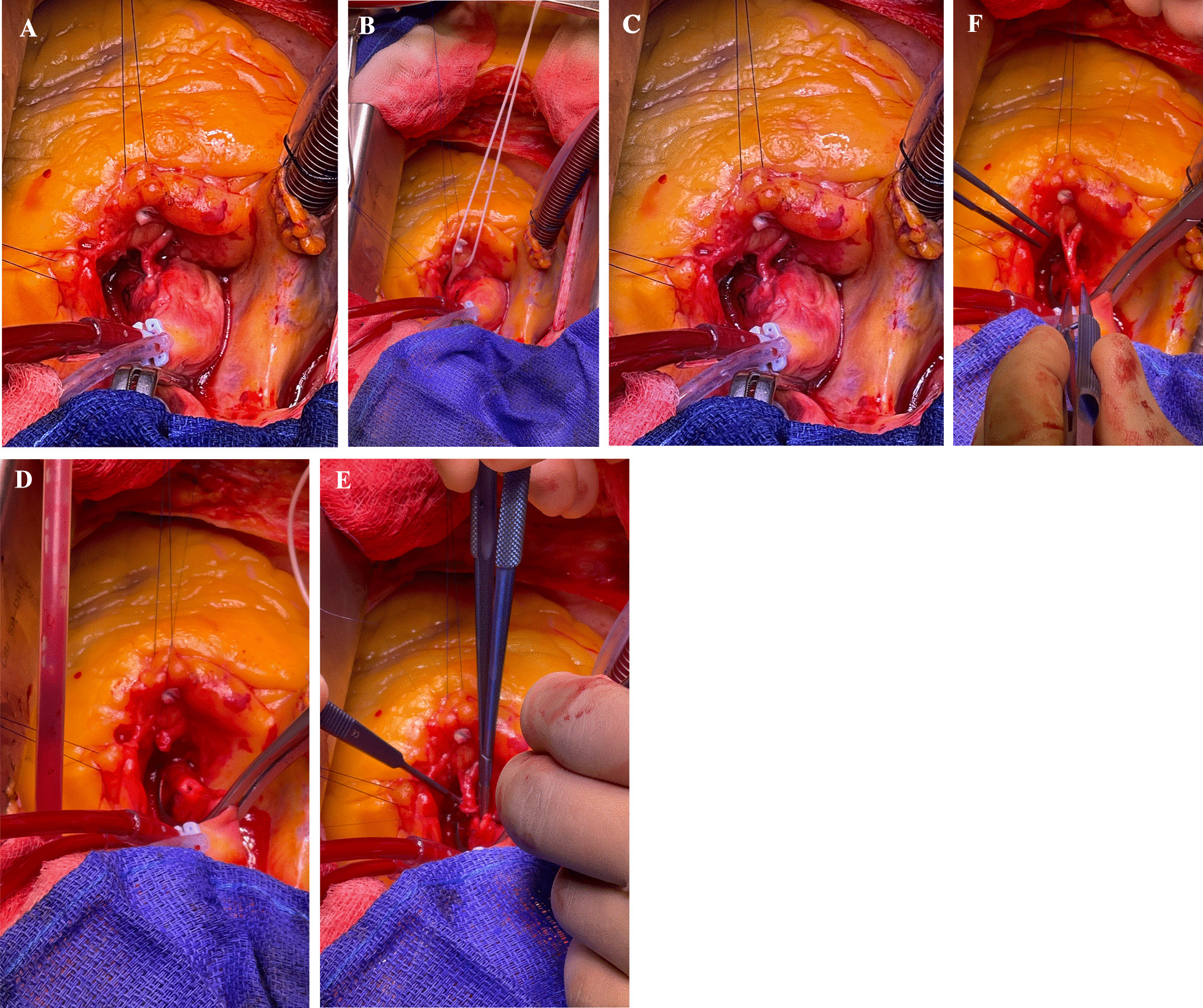
Dissection of the right coronary artery and anastomosis to the aorta. **A** Adequate mobilisation of the right coronary artery distal to its intramural origin (white arrow). **B** Two clips are placed close to the proximal end and the artery is divided, ensuring maximum length available for anastomosis. **C** Right coronary artery clipped as distal as possible, close to intramural origin, to allow for maximum length for anastamosis to the aorta. **D** Right coronary artery divided proximal to the clip. Side biting clamp applied to the aorta. 4.0mm aortic punch used for anastomosis. **E** End-to-side anastomosis performed using 7.0mm prolene. **F** The final tension-free anastomosis

**Fig. 2:**
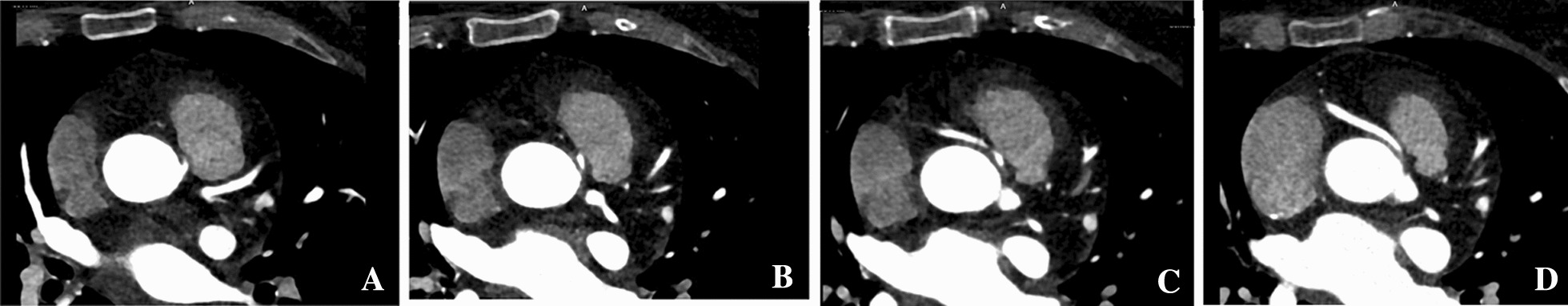
Preoperative computed tomograph cine in axial plane demonstrating the interarterial course of the right coronary artery arising from the opposite sinus in a 68-year old woman presenting with progressively worsening anginal pain. **A** Left coronary artery is visible and right coronary artery is not visible. **B** Origin of right coronary artery visible arising from the aorta between the aorta and pulmonary artery. **C**–**D** Intramural course of right coronary artery

**Fig. 3:**
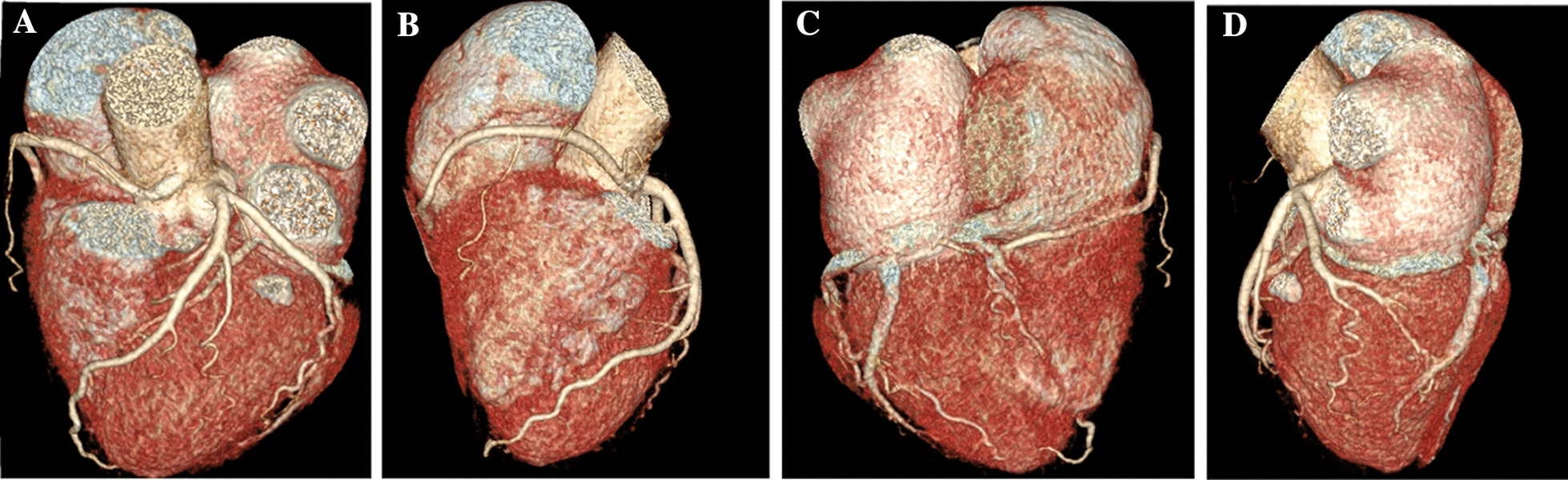
Preoperative  reconstructed 3D computed tomography demonstrating the interarterial course of the right coronary artery. **A** Anterior view showing the origin left main from the left coronary sinus branching into the LAD and LCx. RCA appears to be entering intramurally into the aortic wall. **B** Right view of the heart showing the course of the RCA in the atrial-ventricular groove. **C** Posterior view showing PDA arising from the circumflex. **D** Left view of the heart showing the LMA, LAD, and LCx. RCA appears to become intramural and no longer visible

A decision to proceed with surgical correction was made after discussion at a multidisciplinary meeting. We decided to perform the surgical correction through median sternotomy, on cardiopulmonary bypass, with cardioplegic arrest. The proximal right coronary artery was dissected free from its surrounding tissue from where it appeared to originate from the aortic wall. Care was taken to preserve all the branches. The artery was double clipped just as it exited the aorta, and transected distally. The resulting right coronary artery was anastomosed with a neo-ostium on the aorta created using a 4 mm aortic punch (Medtronic, Minneapolis, Minnesota) (Fig. 1). The patient was weaned off cardiopulmonary bypass and shifted to the ICU (Figs. 2 and 3).

The patient remained in sinus rhythm post-operatively. She was started on dual anti-platelets and beta-blocker from post-op day one. She has an uneventful recovery with no complications and was discharged from the hospital on the seventh postoperative day. Post procedure CT scanning confirmed patency of the relocated right coronary artery.

## Comment

Anomalous origin of right coronary artery (ARCA) from the left sinus of Valsalva is rare with an estimated angiographic prevalence of < 1% [1]. ARCA and anomalous origin of left coronary artery (ALCA) are part of the collective known as Anomalous aortic origin of the coronary artery (AAOCA and associated with sudden cardiac death [2].

With the increasing use of imaging modalities, diagnoses of congenital anomalies of coronary arteries have become relatively common, ranging from 0.3 to 1.3% of the population. Survival to adulthood is rare and dependent on inter-coronary collaterals [3]. Although most cases are clinically insignificant, the highest risks are with anomalous origin from the opposite sinus, particularly with an interarterial course, between the aorta and the pulmonary artery [4].

The management of ARCA remains controversial despite its unpredictable nature, but surgical correction is warranted in all symptomatic patients. The recently published Guidelines for the Management of Adults Congenital Heart Disease places a class I recommendation for revascularization in symptomatic patients with anomalous aortic origin of a coronary artery arising from either the left or the right and a class IIa indication in all asymptomatic patients with evidence of myocardial ischemia. A similar indication is recommended by the 2018 AHA/ACC Guideline for the Management of Adults with Congenital Heart Disease [5, 6]. Although there is limited data available, expert consensus suggests that it is reasonable for adults with this malformation to undergo surgical repair.

Multiple surgical options are available for treating AAOCA, including bypass graft, reimplantation of the anomalous vessels, patch augmentation, pulmonary artery translocation, and unroofing [2]. Surgical unroofing technique was initially established in 1981 and has become the preferred choice for patients with anomalous vessels, particularly coronary arteries with an intramural course or segment [2]. However, surgical unroofing was not appropriate in our patient due to the torturous course of the coronary artery. Additionally, surgical unroofing may cause aortic regurgitation and leave untreated myocardial ischemia in some patients [7]. A second approach considered was using a graft to bypass the culprit artery, using either a saphenous vein graft or an arterial graft. This option was dismissed due to the risks of occlusion of the arterial graft caused by competitive flow, and the possibility of the venous graft failing within the first 5 years, causing the patient to require redo surgery. We chose the reimplantation technique, as it avoided the risks associated with both unroofing and coronary artery bypass grafting. In some cases, reimplantation may require extensive mobilisation to prevent stretching or kinking of the artery. Extensive mobilisation can lead to kinking of the artery and inadequate mobilisation can lead to stretching of the anastomotic site. Reimplantation requires “adequate” mobilisation. We based our revascularisation strategy on the premise that we were able to dissect an adequate length of artery free without sacrificing any branches. This allowed us to maintain a tension-free anastomosis without giving up on the native artery or using a graft that may be prone to occlusion in the future.

Limited literature is available on the non-surgical strategies. Case reports have shown some AAOCA cases being corrected with beta-blockers and/or stents in combination with recommendations for individuals to not participate in competitive or “non-strenuous” activity [2].

In conclusion, we believe that surgical correction of ARCA should be considered in the symptomatic and asymptomatic patient due to its unpredictable nature and the possibility of sudden death. We found that direct implantation of the right coronary artery if the adequate length is obtained to be a safe and reproducible method for surgical correction.

## Data Availability

Not applicable.
